# Supplementary material for: Cine-MRI and T1TSE Sequence for Mediastinal Mass
Source: Cancers (Basel). 2024 Sep 15;16(18):3162. doi: 10.3390/cancers16183162 (PMC11429514; doi:10.3390/cancers16183162)
Supplement: Supplementary file 1 [file cancers-16-03162-s001.zip › Supplementary Figure S1.pdf]

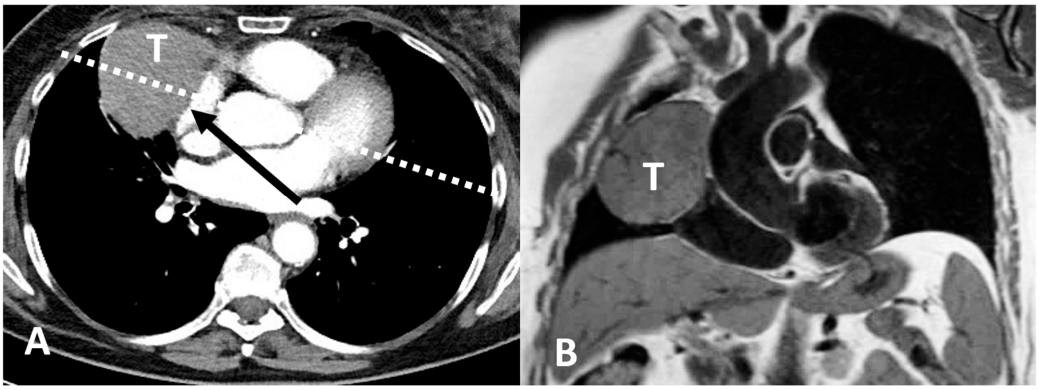

Supplementary Figure S1: False positive CT, true T1TSE

71-year-old female patient with A/B Thymoma (pT1a pN0(0/1) L0 V0 R0, Masaoka-Koga: I) prior to primary tumor resection with RATS. Contrast enhanced CT suspected atrial tumor infiltration (A) which did not confirm in T1TSE sequences due to intervening fat plane (B), free structure motion in cine-MRI (supplementary videos 1 and 2) and intraoperatively.

RATS = robotic-assisted thoracic surgery, CT = computed tomography, T1TSE = magnetic resonance imaging (MRI)/T1-weighted spin echo sequence, cine-MRI = cine magnetic resonance imaging, T = tumor

(A) Axial contrast enhanced CT with suspected atrial tumor infiltration (black arrow)

(B) Paracoronal plane perpendicular to the plane suspected for infiltration (white dashed line in (A)): atrial tumor infiltration not suspected (true negative) due to a prominent fat lamella.
