# Supplementary material for: Cine-MRI and T1TSE Sequence for Mediastinal Mass
Source: Cancers (Basel). 2024 Sep 15;16(18):3162. doi: 10.3390/cancers16183162 (PMC11429514; doi:10.3390/cancers16183162)
Supplement: Supplementary file 1 [file cancers-16-03162-s001.zip › Supplementary Figure S2.pdf]

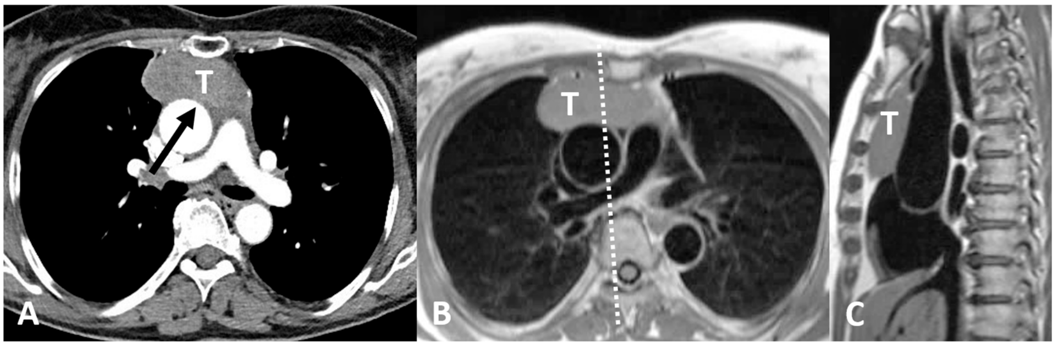

Supplementary Figure S2: Unclear CT, correct cine-MRI

61-year-old female patient with B2 thymoma (pT1a pN0 (0/3) L0 V0 R0, Masaoka-Koga: I) prior to primary tumor resection with RATS. Contrast enhanced CT diagnosis was unclear (A). Due to missing intervening fat plane T1TSE sequence diagnosis was also unclear (B) and (C). Additional cine-MRI did not reveal aortic infiltration (true negative, supplementary material videos 3 and 4). RATS = robotic-assisted thoracic surgery, CT = computed tomography, T1TSE = magnetic resonance imaging (MRI)/T1-weighted spin echo sequences, cine-MRI = cine magnetic resonance imaging, T = tumor

(A) Axial contrast enhanced CT with unclear diagnosis concerning aortic tumor infiltration (black arrow)

(B) Axial T1TSE sequence with unclear aortic tumor infiltration

(C) Parasagittal T1TSE sequence with unclear aortic tumor infiltration (plane perpendicular to the plane suspected for infiltration (white dashed line in (B))
