# Supplementary material for: Cine-MRI and T1TSE Sequence for Mediastinal Mass
Source: Cancers (Basel). 2024 Sep 15;16(18):3162. doi: 10.3390/cancers16183162 (PMC11429514; doi:10.3390/cancers16183162)
Supplement: Supplementary file 1 [file cancers-16-03162-s001.zip › Supplementary Table S1.pdf]

**Modality/sequence Anatomical structure**	**Histology/ Intraoperative Finding (gold standard)**	**CT**	**cine-MRI**	**T1TSE**
Pericardium	yes/no	yes/no/unclear	yes/no/unclear	yes/no/unclear
Myocardium	yes/no	yes/no/unclear	yes/no/unclear	yes/no/unclear
SVC	yes/no	yes/no/unclear	yes/no/unclear	yes/no/unclear
Aorta	yes/no	yes/no/unclear	yes/no/unclear	yes/no/unclear
Pulmonary arteries	yes/no	yes/no/unclear	yes/no/unclear	yes/no/unclear
Atria	yes/no	yes/no/unclear	yes/no/unclear	yes/no/unclear

Supplementary Table S1: Score sheet for cine-MRI, T1TSE and CT evaluation.cine-MRI = cine magnetic resonance imaging. T1TSE = magnetic resonance imaging (MRI)/T1-weighted spin echo sequences. CT = computed tomography. SVC = superior vena cava
