# Supplementary material for: Cine-MRI and T1TSE Sequence for Mediastinal Mass
Source: Cancers (Basel). 2024 Sep 15;16(18):3162. doi: 10.3390/cancers16183162 (PMC11429514; doi:10.3390/cancers16183162)
Supplement: Supplementary file 1 [file cancers-16-03162-s001.zip › Supplementary Table S2.pdf]

	Gold standard	CT				
	Infiltration: Yes/No	True positive	True negative	False positive	False negative	Unclear
Pericardium	19/28	8	12	12	1	14
Myocardium	0/47	0	44	1	0	2
SVC	1/46	0	43	2	0	2
Aorta	2/45	1	22	2	1	21
Pulmonary arteries	0/47	0	32	2	0	13
Atria	0/47	0	36	6	0	5

Supplementary Table S2: Synopsis of radiologic CT evaluation matched to gold standard. CT = computed tomography, SVC = superior vena cava.
