# Supplementary material for: Cine-MRI and T1TSE Sequence for Mediastinal Mass
Source: Cancers (Basel). 2024 Sep 15;16(18):3162. doi: 10.3390/cancers16183162 (PMC11429514; doi:10.3390/cancers16183162)
Supplement: Supplementary file 1 [file cancers-16-03162-s001.zip › Supplementary Table S3.pdf]

	**Gold standard**	**cine MRI**				
	Infiltration: Yes/No	True positive	True negative	False positive	False negative	Unclear
Pericardium	19/28	11	16	9	5	6
Myocardium	0/47	0	44	1	0	2
SVC	1/46	0	42	2	1	2
Aorta	2/45	1	42	3	1	0
Pulmonary arteries	0/47	0	42	2	0	3
Atria	0/47	0	44	2	0	1

Supplementary Table S3: Synopsis of radiologic cine-MRI evaluation matched to gold standard. Cine-MRI = cine magnetic resonance imaging, SVC = superior vena cava.
