# Supplementary material for: Cine-MRI and T1TSE Sequence for Mediastinal Mass
Source: Cancers (Basel). 2024 Sep 15;16(18):3162. doi: 10.3390/cancers16183162 (PMC11429514; doi:10.3390/cancers16183162)
Supplement: Supplementary file 1 [file cancers-16-03162-s001.zip › Supplementary Table S4.pdf]

	**Gold standard**	**T1TSE**				
	Infiltration: Yes/No	True positive	True negative	False positive	False negative	Unclear
Pericardium	19/28	11	19	4	2	11
Myocardium	0/47	0	45	1	0	1
SVC	1/46	0	40	2	0	5
Aorta	2/45	1	25	4	0	17
Pulmonary arteries	0/47	0	37	2	0	8
Atria	0/47	0	38	3	0	6

Supplementary Table S4: Synopsis of radiologic T1TSE sequence evaluation matched to gold standard. T1TSE = magnetic resonance imaging (MRI)/T1-weighted spin echo se-quences, SVC = superior vena cava.
