# Supplementary material for: Cine-MRI and T1TSE Sequence for Mediastinal Mass
Source: Cancers (Basel). 2024 Sep 15;16(18):3162. doi: 10.3390/cancers16183162 (PMC11429514; doi:10.3390/cancers16183162)
Supplement: Supplementary file 1 [file cancers-16-03162-s001.zip › Supplementary Video S1 Caption.pdf]

Supplementary Video S1 – False positive CT, true positive T1TSE

T1TSE sequence of a 71-year-old female patient (see also supplementary figure 1) with free cardiac motion and no signs of atrial tumor infiltration. CT = computed tomography; T1TSE = magnetic resonance imaging (MRI)/T1-weighted spin echo sequences
