# Supplementary material for: Cine-MRI and T1TSE Sequence for Mediastinal Mass
Source: Cancers (Basel). 2024 Sep 15;16(18):3162. doi: 10.3390/cancers16183162 (PMC11429514; doi:10.3390/cancers16183162)
Supplement: Supplementary file 1 [file cancers-16-03162-s001.zip › Supplementary Video S4 Caption.pdf]

Supplementary Video S4: Unclear CT, correct cine-MRI

Cine-MRI of 61-year-old female patient (see also supplementary figure 2) with suspected aortic infiltration in computed tomography and unclear T1TSE sequence. Cine-MRI shows free cardiac motion without signs of aortic infiltration. CT = computed tomography; cine-MRI = cine magnetic resonance imaging, T1TSE = magnetic resonance imaging (MRI)/T1-weighted spin echo sequences.
